# Prevalence and determinants of depression in mothers of children under 5 years in Bole District, Ghana

**DOI:** 10.1186/s13104-019-4399-5

**Published:** 2019-07-01

**Authors:** Nawaf Saeed, Anthony Wemakor

**Affiliations:** School of Allied Health Sciences, University for Development Studies, Tamale, Ghana

**Keywords:** Depression, Mothers, Firewood, Marital status, Occupation, Tamale

## Abstract

**Objective:**

Depression in mothers is a risk factor for poor health, and pregnancy and child outcomes. The objective of the present study was to determine the prevalence of depression and identify its determinants in mothers of children under 5 years in Bole District, Ghana.

**Results:**

We conducted an analytical cross-sectional study consisting of 244 mothers (mean age 28.7 ± 6.29 years) in Bole District, Ghana. Edinburgh Postnatal Depression Scale was used to screen for depression and the determinants of depression were identified using logistic regression analysis. The prevalence of depression in this study population was 16.8% (95% confidence interval 12.1–22.0%). The independent determinants of depression were marital status, occupation, lighting source and type of cooking fuel. Being currently unmarried (p < 0.001), and using lighting sources other than electricity (p = 0.004) were associated with higher risk of depression while being employed in other occupations (p = 0.001), and not cooking with firewood (p = 0.008) were associated with lower risk of depression. In this study population, the prevalence of depression was relatively high in mothers and was associated with marital status, occupation, lighting source and cooking fuel. Interventions to prevent and treat depression in women should include strategies to improve their socio-economic status and living conditions.

## Introduction

Depression is common in women of childbearing age [1]. In the developed world, 10 to 16% of all mothers are estimated to experience depression [2] while in Africa, a review based on 35 studies estimated that 18.3% of mothers have depression [3]. In Northern Ghana, the prevalence of depression was estimated in two community-based studies as 27.8% in 2016 [4] and 33.5 in 2018 [5] while six studies conducted on mothers outside northern Ghana recorded lower rates (3.8–11.3%) [6–11]. Depression is the leading cause of morbidity and mortality worldwide [12].

Maternal depression can encourage poor health behaviour and increase the risk of unfavourable birth and child outcomes [13]. This includes preterm birth, low birth weight, and reduced growth and psychological problems [14, 15]. Untreated depression has been linked to the development of maladjustment in children [16] and poor psychological and physical wellbeing of children and adolescents [17].

Research in low-resourced settings has shown an association between maternal mental health and caregiving practice, and this has implications for the health and well-being of children. Studies on mothers with depressive symptoms in Ghana and Ivory Coast showed high prevalence of diarrhoeal and febrile diseases in children of depressed mothers compared to children of psychological sound mothers [11]. Additionally, maternal depression in both high- and low-income countries has been shown to negatively affect breastfeeding [18, 19]. Some studies have found that mothers with depression stopped exclusive breastfeeding early compared to those without depressive symptoms and other studies found that depressive mothers were more likely to stop breastfeeding entirely in the complementary feeding period [20, 21].

In spite of the link between maternal mental health and pregnancy and child outcomes, data are scanty on the prevalence and determinants of depression in mothers of young children in Ghana. The objective of the present study was to determine the prevalence of depression and identify its determinants in mothers of children under 5 years in Bole District, Ghana.

## Main text

### Methods

#### Study design, area and population

An analytical cross-sectional study was conducted in Mankuma in Bole District, Ghana, in February, 2015. The Bole District is located at the extreme western part of the Savanna Region, one of the 16 administrative regions of Ghana. The study population consisted of all mothers of children under 5 years living in the Mankuma community.

#### Sample size determination and sampling

Using the single population proportion formula [22], a sample size of 244 was estimated. A list of all mothers of children under 5 years in the Mankuma community was compiled using Child Welfare Records with the help of community health volunteers. Simple random sampling was used in selecting the 244 mothers who were visited and interviewed at home.

#### Data collection

A researcher-constructed semi-structured questionnaire, and Edinburgh Postnatal Depression Scale (EPDS) were used to collect data for the study. The semi-structured questionnaire has sections on socio-demographic and economic characteristics, and household cooking fuel and lighting sources. These variables were assessed in our study because previous studies have reported their association with mental health status of women in developing countries [23–25]. The EPDS was developed for screening postpartum women in outpatient, home visit settings, or at the 6–8 weeks postpartum examination [26]. The tool has been used among numerous populations in various countries. The EPDS is made up of 10 questions. Responses are scored 0, 1, 2, or 3 according to increased severity of the symptoms for questions 1, 2 and 4. Questions 3, and 5–10 are reverse scored (i.e., 3, 2, 1, and 0). The total score is obtained by adding together the scores for each of the 10 items, which gives a maximum score of 30. The cut-off used to define depression is 12, thus, a woman scoring 12 or more points was classified as being depressed [27]. The administration of the questionnaires was undertaken by 3 final year undergraduate students of the School of Allied Health Sciences, University for Development Studies, Tamale. The questionnaires were administered in Gonja in face-to-face interviews with the mothers.

#### Statistical analysis

Data were analyzed using Stata IC (Version 12.1). Means and standard deviations were calculated for continuous variables and frequencies and percentages for categorical variables. Household wealth index was derived using 12 household items i.e., radio, television, satellite dish, sewing machine, mattress, refrigerator, DVD, electric fan, mobile phone, bicycle, motorcycle and car following a standard procedure [28]. Possession of each of the items attracted a score of 1 otherwise a score of 0. Using Principal Component Analysis, household wealth scores were derived for each household, and all the scores were ranked and divided into 3 equal groups: poorest, medium and richest. This index is considered a good proxy for socio-economic status and is routinely reported in the Ghana Demographic and Health Survey reports. Chi-square or Fisher’s exact test was employed in the bivariate analyses and variables that were significant (p < 0.2) in these analyses were considered for multivariate logistic regression analysis. Declaration of significance in the multivariate analysis was done at p < 0.05.

### Results

Majority of our participants were currently married (84.0%), belonged to the Islamic faith (77.0%) and were from Gonja tribe (73.0%). Most of them (43.0%) were traders, and the majority had no education (53.3%). Again, the majority of them had one child under 5 (70.1%), used electricity as a source of lighting (95.5%) and used firewood for cooking (70.5%) (Table 1).

**Table 1:** Socio-demographic characteristics (*n *= 244)

Characteristic	Frequency (n)	Percent
Age group (years)		
18–19	14	5.7
20–24	48	19.7
25–29	82	33.6
30–34	58	23.8
35–39	24	9.8
40 +	18	7.4
Education		
None	130	53.3
Primary	55	22.5
Junior High School and above	59	24.2
Marital status^a^		
Currently married	205	84.0
Currently unmarried	39	16.0
Religion		
Islam	188	77.0
Others	56	23.0
Ethnicity		
Gonja	178	73.0
Others	66	27.0
Occupation		
Trader/vendor	105	43.0
Housewife	53	21.7
Others	86	35.2
Number of children under 5 in household		
1	171	70.1
2	73	29.9
Source of lighting in the house		
Electricity	233	95.5
Others	11	4.5
Type of fuel used for cooking		
Firewood	172	70.5
Others	72	29.5
Household wealth index (tertile)		
Poorest	84	34.4
Medium	81	33.2
Richest	79	32.4

^a^Legally married women were classified as “currently married”, all other women were classified as “currently unmarried”

#### Maternal depression and its determinants

The prevalence of maternal depression was estimated at 16.8% (95% confidence interval: 12.1–22.0%) for the study subjects. In bivariate analyses, 8 variables had crude associations with depression status of the mothers namely: age (p = 0.027), marital status (p < 0.001), religion (p = 0.062), ethnicity (p = 0.058), occupations (p = 0.061), source of lighting in the house (p < 0.001), type of fuel used for cooking (p = 0.056), and household wealth index (p = 0.015) (Table 2). These variables were entered into a logistic regression model and after mutual adjustment using stepwise logistic regression procedure, marital status, occupation, source of lighting in the household, and type of fuel used for cooking remain associated with maternal depression independently. Being currently unmarried (p < 0.001), and using lighting sources other than electricity (p = 0.004) were associated with higher risk of depression while being employed in other occupations (mostly farming and provision of services) (p = 0.001) and not cooking with firewood (p = 0.008) were associated with lower risk of depression (Table 3).

**Table 2:** Comparison of depressed and non-depressed mothers (*n *= 244)

Characteristic	Total	Depression status		Test statistics
		No, n (%)	Yes, n (%)	
Age group (years)				
18–24	62	45 (72.6)	17 (27.4)	Chi-square = 7.223; df = 2; p = 0.027
25–29	82	73 (89.0)	9 (11.0)	
30 +	100	85 (85.0)	15 (15.0)	
Education				
None	130	111 (85.4)	19 (14.6)	Chi-square = 1.435; df = 2; p = 0.488
Primary	55	43 (78.2)	12 (21.8)	
JHS and above	59	49 (83.1)	10 (16.9)	
Marital status^a^				
Currently married	205	179 (87.3)	26 (12.7)	Chi-square = 15.576; df = 1; p < 0.001
Currently unmarried	39	24 (61.5)	15 (38.5)	
Religion				
Islam	188	161 (85.6)	27 (14.4)	Chi-square = 3.493; df = 1; p = 0.062
Others	56	42 (75.0)	14 (25.0)	
Ethnicity				
Gonja	178	153 (86.0)	25 (14.0)	Chi-square = 3.581; df = 1; p = 0.058
Others	66	50 (75.8)	16 (24.2)	
Occupation				
Trader/vendor	105	82 (78.1)	23 (21.9)	Chi-square = 5.578; df = 2; p = 0.061
Housewife	53	43 (81.1)	10 (18.9)	
Others^b^	86	78 (90.7)	8 (9.3)	
Number of children under 5 in household				
1	171	140 (81.9)	31 (18.1)	Chi-square = 0.718; df = 1; p = 0.397
2	73	63 (86.3)	10 (13.7)	
Source of lighting in the house				
Electricity	233	199 (85.4)	34 (14.6)	p < 0.001^c^
Others	11	4 (36.4)	7 (63.6)	
Type of fuel used for cooking				
Firewood	172	138 (80.2)	34 (19.8)	Chi-square = 3.663; df = 1; p = 0.056
Others	72	65 (90.3)	7 (9.7)	
Household wealth index (tertile)				
Lowest	84	62 (73.8)	22 (26.2)	Chi-square = 8.416; df = 2; p = 0.015
Medium	81	70 (86.4)	11 (13.6)	
Highest	79	71 (89.9)	8 (10.1)	

^a^Legally married were classified as “currently married”, all other women were classified as “currently unmarried”

^b^Includes agriculture workers (n = 45), services providers (n = 34) and salaried workers (n = 7)

^c^Fisher’s exact test used for comparison

**Table 3:** Multivariate analysis of the determinants of depression in mothers (*n *= 244)

Characteristic	Adjusted OR	95% confidence interval	*p* value
Marital status^a^			
Currently married	1.00		
Currently unmarried	5.24	2.06–13.28	< 0.001
Occupation			
Trader/vendor	1.00		
Housewife	0.38	0.14–1.06	0.063
Others^b^	0.18	0.06–0.51	0.001
Ethnicity			
Gonja	1.00		
Others	2.32	1.02–5.28	0.046
Source of lighting in the household			
Electricity	1.00		
Others	8.72	2.00–38.08	0.004
Type of fuel used for cooking			
Firewood	1.00		
Others	0.23	0.08–0.68	0.008

^a^Legally married women were classified as “currently married”, all other women were classified as “currently unmarried”

^b^This includes mainly agriculture workers (n = 45) and services providers (n = 34) and a few salaried workers (n = 7)

### Discussion

The present study found the prevalence of depression to be 16.8% among mothers of children under 5 years in Mankuma in Bole District, Ghana. Among our sampled population, the independent determinants of depression are marital status, occupation, lighting source and cooking fuel.

A lower prevalence of maternal depression was estimated than previously reported for mothers in Northern Ghana. Two previous studies, both of which used the Centre for Epidemiologic Studies Depression Scale, reported about twice our rate in Northern Ghana mothers i.e., 27.8% in 2016 [4] and 33.5% in 2018 [5]. However, six studies conducted on mothers outside northern Ghana recorded lower rates (3.8–11.3%) [6–11]. The lack of a nationally representative study on the prevalence of depression in Ghana makes judging these figures difficult. It is possible that study population, screening tool, study design and other factors explain the disparities in the rates of depression in mothers in Ghana.

We found that marital status, occupation, household lighting and cooking fuel were independently associated with depression status of the mothers. The finding of lower risk of depression for married women was supported by some studies [29, 30]. A similar study to ours has shown that married mothers have lower chances of being depressed compared to unmarried ones especially if their husbands are literate and caring [29]. The authors attributed the higher risk of depression associated with unmarried status to lack of partners to confide in and share one’s woes with and this is likely to be the situation for our study population also.

We however found a lower risk of depression for women who engaged in other occupations (mostly farming and provision of services) relative to trading. The exact reasons are not known but this may relate to the stress associated with the difficult economic situation in Ghana arising from high interest rates on bank loans [31], and the constant depreciation of the Ghanaian currency (Ghana Cedis) against the US dollar [32]. This is likely to impact traders more severely compared to people in other occupations as they need to take bank loans to run their businesses, and the US dollar is needed for importing goods. The rapid depreciation of the Ghana Cedis also means that the prices of goods keep going up and this affects sales and leads to lower return on investments.

We found an increased risk for depression for women in households not using electricity for lighting but these women were very few (4.5% of total sample). Mothers who used other fuels were 77% less likely to be depressed compared to those who used firewood for cooking and a similar study to ours found higher rates of depression in women who used biomass for cooking compared to other fuels in India [24]. The use of firewood for cooking connotes underdevelopment and low socio-economic status as it is used by more than 65.0% of the population in rural areas of Ghana who are mostly poor [33] and only 5.5% of this population use liquefied petroleum gas as their main cooking fuel [34]. The mechanism by which firewood use increases the risk of depression is not known but may relate to the stress involved in gathering it from the field and the smoke associated with its use.

### Conclusions

The current study found a relatively high prevalence of depression (16.8%) in mothers of children under 5 years in Bole District, Ghana. The socio-demographic factors associated with depression were marital status, occupation, lighting source and type of cooking fuel. Interventions to prevent and treat depression in women should include strategies to improve their socio-economic status and living conditions.

## Limitation

One major limitation with our study is that, the EPDS used to determine depression status was not validated for our study population so its reliability in this context cannot be judged. Despite this limitation, this study provides significant insights into the level of depression and its determinants in mothers of children under 5 years of age in the Bole District, Ghana.

## Data Availability

The minimal dataset analysed during the current study can be obtained from the corresponding author upon reasonable request.

## Abbreviations

AOR: adjusted odds ratio
CI: confidence interval
EPDS: Edinburgh Postnatal Depression Scale
